# Functional Cure of SIVagm Infection in Rhesus Macaques Results in Complete Recovery of CD4^+^ T Cells and Is Reverted by CD8^+^ Cell Depletion

**DOI:** 10.1371/journal.ppat.1002170

**Published:** 2011-08-04

**Authors:** Ivona Pandrea, Thaidra Gaufin, Rajeev Gautam, Jan Kristoff, Daniel Mandell, David Montefiori, Brandon F. Keele, Ruy M. Ribeiro, Ronald S. Veazey, Cristian Apetrei

**Affiliations:** 1 Division of Comparative Pathology, Tulane National Primate Research Center, Covington, Louisiana, United States of America; 2 Department of Pathology, School of Medicine, University of Pittsburgh, Pittsburgh, Pennsylvania, United States of America; 3 Center for Vaccine Research, University of Pittsburgh, Pittsburgh, Pennsylvania, United States of America; 4 Division of Microbiology, Tulane National Primate Research Center, Covington, Louisiana, United States of America; 5 Department of Surgery, Duke University, Durham, North Carolina, United States of America; 6 SAIC Frederick, Inc, NCI, NIH, Frederick, Maryland, United States of America; 7 Los Alamos National Laboratory, Los Alamos New Mexico, United States of America; 8 Department of Microbiology and Molecular Genetics, School of Medicine, University of Pittsburgh, Pittsburgh, Pennsylvania, United States of America; Harvard University, United States of America

## Abstract

Understanding the mechanism of infection control in elite controllers (EC) may shed light on the correlates of control of disease progression in HIV infection. However, limitations have prevented a clear understanding of the mechanisms of elite controlled infection, as these studies can only be performed at randomly selected late time points in infection, after control is achieved, and the access to tissues is limited. We report that SIVagm infection is elite-controlled in rhesus macaques (RMs) and therefore can be used as an animal model for EC HIV infection. A robust acute infection, with high levels of viral replication and dramatic mucosal CD4^+^ T cell depletion, similar to pathogenic HIV-1/SIV infections of humans and RMs, was followed by complete and durable control of SIVagm replication, defined as: undetectable VLs in blood and tissues beginning 72 to 90 days postinoculation (pi) and continuing at least 4 years; seroreversion; progressive recovery of mucosal CD4^+^ T cells, with complete recovery by 4 years pi; normal levels of T cell immune activation, proliferation, and apoptosis; and no disease progression. This “functional cure” of SIVagm infection in RMs could be reverted after 4 years of control of infection by depleting CD8 cells, which resulted in transient rebounds of VLs, thus suggesting that control may be at least in part immune mediated. Viral control was independent of MHC, partial APOBEC restriction was not involved in SIVagm control in RMs and Trim5 genotypes did not impact viral replication. This new animal model of EC lentiviral infection, in which complete control can be predicted in all cases, permits research on the early events of infection in blood and tissues, before the defining characteristics of EC are evident and when host factors are actively driving the infection towards the EC status.

## Author Summary

A small proportion of HIV-infected patients control viral replication and disease progression in the absence of any antiretroviral treatment. Understanding the mechanisms of viral control in these elite controllers may help to identify new therapeutic approaches in order to control HIV infection. However, elite controllers are identified AFTER control is established, therefore it is difficult to identify the virus and host factors that drive the infection to the controlled status. We identified an animal model (the rhesus macaque infection with SIVagm) in which, after massive acute viral replication and CD4+ T cell depletion, SIV infection is controlled in 100% of cases during chronic infection. This “functional cure” of SIVagm infection in rhesus macaques results in a complete immune restoration after four years and can be reverted by depleting the cellular immune responses *in vivo*. An animal model of elite controlled lentiviral infection in which complete control can be predicted in all cases permits research on the early events of infection when host factors are actively driving the infection towards the controlled status to understand the pathogenesis of HIV/SIV infections and design of new approaches for controlling HIV infection.

## Introduction

A minority of HIV-1-infected patients, defined as elite controllers (ECs), demonstrate that effective control of viral replication and disease progression is possible in the absence of treatment [1]. Deciphering the mechanisms of natural infection control in these patients is a critical step for designing successful vaccines and is considered a priority in the field [2]. Research on human ECs, however, is restricted to studies of the chronic infection, samples collected at random time points, and limited access to tissues, all precluding identification of early events responsible for virus control. These shortcomings have likely prevented a clear understanding of the immune mechanisms driving infection towards elite-controlled status. An animal model in which complete control of viral replication can be achieved in all cases has strong potential to effectively complement EC research in humans.

Pathogenic HIV and SIV infections of humans and RMs are characterized by progression to AIDS in a variable time frame [3] and are associated with: (i) massive, continuous viral replication [4], [5], with VL set-points being predictive for the time of progression to AIDS [6]; (ii) continuous depletion of CD4^+^ T cells in peripheral blood [7] which is more pronounced at mucosal sites [8], [9], [10], and (iii) high levels of T cell immune activation [11], the magnitude of which has been reported to be predictive of disease progression [11]; (iv) only partial control of viral replication through immune responses [12]. The interaction among these factors cripples the immune system and eventually results in severe immunodeficiency and death [7]. Antiretroviral (ARV) treatments have improved survival, but they do not always provide complete control of viral replication [13] and are plagued by the issues of ARV resistance and multiple side effects, which limit their long-term use [14]. On the other hand, effective HIV/SIV vaccines are not yet available to prevent the spread of HIV [15], [16].

A fraction of HIV-infected patients (1–5%) are long-term nonprogressors (LTNP), with ECs being a subset of long-term nonprogressors. The main characteristics of LTNP infections are (i) infection for more than 7 years; (ii) stable CD4^+^ T cell counts greater than 600 cells/µl; (iii) low/undetectable levels of HIV in the peripheral blood; (iv) no symptoms of HIV-induced disease; (v) apparent control of viral replication through vigorous CD4^+^ and CD8^+^ T cell responses against HIV suggesting, but not proving that these cells may be causally related to virus control [1]. These vigorous immune responses against HIV are characterized by multifunctional, persistent CD8 responses, vigorous HIV-specific interferon (IFN)-γ and antiviral β-chemokines (including RANTES), CD4^+^ T cell responses and macrophage inflammatory protein MIP-1α and MIP-1β responses [17]; and (vi) lower expression of PD-1 and CTLA-4 in LTNPs than in normal progressors, with the ECs having the least expression [18]. All these features occur in the absence of any ARV therapy. Similarly, a minority of SIVmac-infected RMs is characterized by better control of SIV infection, and these are also referred to as ECs [19], [20].

Some EC infections may either be intrinsic to the infecting viral strain (i.e., nef-defective strains in humans or RMs), or host (i.e., heterozygosity for the *CCR5Δ32* allele). However, the most relevant category for understanding the mechanisms of protection in EC infection is the one in which control is achieved through effective host responses. There is a consistent association between certain class I alleles and EC status, however the mechanistic role of some of these alleles (i.e., HLA-B5701) in the control of HIV remains an open question [1]. Conversely, viral control is immune-mediated in human EC infections associated with B27 allele, or those associated with B*08 allele in RMs [1]. Finally, about 40% of both human and RM ECs have no identified host genetic traits associated with viral control [1]. Therefore, our understanding of the mechanisms of EC infection would greatly benefit from the possibility to study an EC infection before the control is actually achieved, but when factors driving infection to EC status are likely acting. The mechanisms underlying the spontaneous control of SIV infection in ECs can provide clues for the design of effective vaccine strategies or for the development of a functional cure of HIV infection (defined by complete and durable control of the HIV infection in the absence of virus eradication).

Here we report the development of an animal model of elite controlled infection in which control occurs in 100% of cases and thus can be predicted at the stages of infection in which the virus is still actively replicating. This animal model is based on RM infection with SIVagm.sab, which is characterized by robust acute viral replication and immune activation, massive acute mucosal CD4^+^ T cell depletion, followed by complete control of viral replication during the chronic stage, which results in complete recovery of immunologic injuries inflicted during the acute infection. We also report that complete control of SIVagm infection in RMs can be reversed following CD8 depletion *in vivo*, demonstrating persistence of the SIVagm in RMs and suggesting control through immune responses. This new animal model of “super elite” controlled infection can be use to understand a functional cure of HIV infection.

## Results

### SIVagm infection of RMs

Twelve RMs were intravenously infected with 100 TCID50 of SIVagm.sab92018 directly derived from an acutely-infected AGM [21]. Four of them were followed for more than 6 years post-infection (p.i.). Two RMs were followed for 450 days p.i., while the remaining RMs were serially sacrificed during acute and early chronic infection. During the acute infection, RMs infected with SIVagm showed significant lymphadenopathy. One RM developed a rash, while 6 of them experienced weight loss and fever. However, after three months, SIVagm-infected RMs controlled viral replication (see below) and none of them showed any clinical or biological signs of disease progression during the follow-up.

### SIVagm.sab replication is controlled in 100% of infected RMs and control is not due to an impairment of viral replication

**Figure 1:**
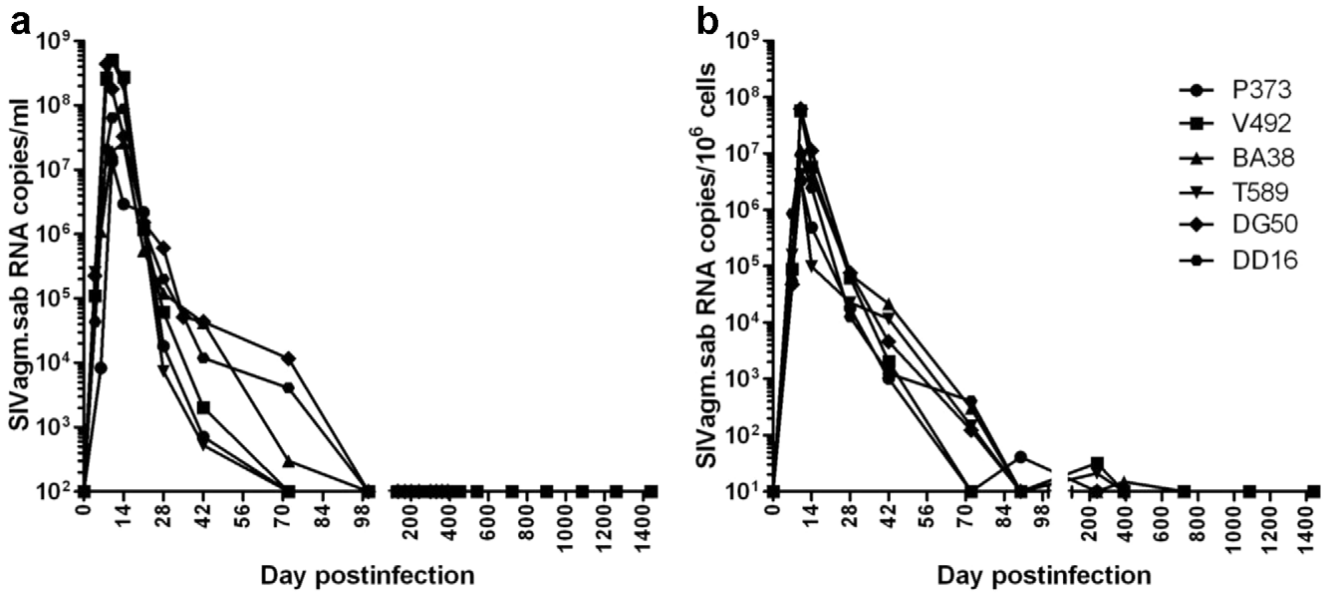
Viral replication during acute and chronic SIVagm infection of RMs. (a) Plasma viral load quantification showed high levels of viral replication during acute infection followed by complete control of viremia, with undetectable plasma viral loads, starting from days 72–98 p.i. on. (b) the overall pattern of SIVagm replication in the intestine generally paralleled that of plasma viral load, but control of viral replication was achieved at later time points (after day 132 p.i.). The detection limit of the assay was 10^2^ copies/ml of plasma and 10 copies per 10^6^ cells.

SIVagm.sab showed active replication in RMs during primary infection, with peak plasma VLs (10^7^–10^9^ copies/ml) occurring by day 10 p.i. (Figure 1a). These VL levels are in the range of those reported for pathogenic SIVmac and HIV-1 infections in RMs and humans, respectively. In contrast to the high viral replication during the acute infection, the postpeak SIVagm.sab VLs continuously declined in RMs until VLs became undetectable, by days 72–98 p.i. and remained so during the follow-up (Figure 1a). SIVagm RNA VL dynamics in the intestine generally paralleled that observed in plasma (Figure 1b). The high levels of viral replication in the intestine during the acute SIVagm infection of RMs were confirmed by *in situ* hybridization (Figure S1). Blips of very low levels of viral replication could be documented at mucosal sites during the first stages of chronic infection, up to day 400 p.i. (Figure 1b). Thus, SIVagm.sab replication in RMs during chronic infection is clearly different from both the replication patterns described in pathogenic SIV/HIV infections (where set-point VLs are established that have predictive values for the duration of disease progression, and increases in VLs occur with disease progression) and in nonpathogenic natural infections of African NHPs (where set-point VLs are maintained indefinitely) [22], [23].

We concluded that (i) the undetectable VLs that last up to 4 years define 100% of SIVagm-infected RMs as ECs; and (ii) SIVagm.sab replication is not restricted in RMs and its control during the late stages of SIV infection is not due to the inability of the virus to replicate in RMs.

**Figure 2:**
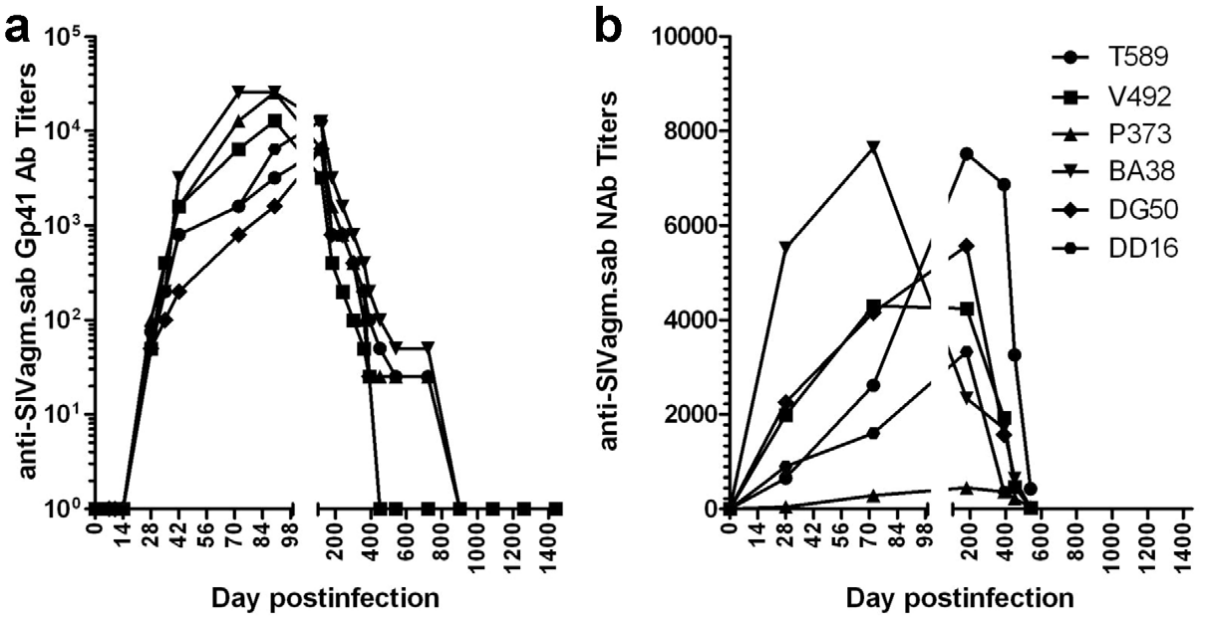
Control of SIVagm replication in RMs was confirmed by seroreversion of anti-SIVagm binding and neutralizing antibodies. (a) Dynamics of anti-gp41 antibody titers in SIVagm-infected RMs by SIVagm.sab-specific ELISA. All animals seroconverted between days 14–21 post-infection. Antibody titers reached the highest levels by day 98 p.i., then continuously declined as VLs were controlled. (b) Neutralizing antibodies against SIVagm.sab92018 also vane with the control of viral replication.

The complete control of SIVagm replication in chronically-infected RMs was further suggested by the dynamics of anti-gp41 SIVagm antibodies in RMs, as detected by a specific SIVagm.sab peptide mapping the immunodominant epitope of gp41 using an in-house ELISA [21]. Seroconversion occurred in all animals by day 21 p.i. (Figure 2a). With the complete control of viral replication during chronic infection, the animals seroreverted starting from day 360 p.i. (Figure 2a). Anti-SIVagm.sab neutralizing antibodies showed a similar dynamics (Figure 2b), also supporting the control of viral replication. The levels of anti-SIVagm.sab92018 in RMs were not substantially different from those observed in experimentally-infected AGMs [24]. Both the magnitude and the dynamics of NAbs suggest that humoral responses are peripheral to the control of SIVagm.sab in rhesus macaques.

Finally, nested PCR for different SIV genomic regions on RNA and DNA from serial samples of plasma, PBMCs, LNs or intestinal lymphocytes collected during chronic infection (days 720, 1080 and 1260 p.i.) were all negative, suggesting the complete control of SIVagm replication by RMs. However, the negative PCRs on these late time point samples may be due to the fact that the PCRs were carried out on a limited number of cells (in general, less than 5×10^5^ cells per assay), which might have been insufficient for the detection of very low amounts of SIVagm.

### Control of SIVagm infection in RMs does not result from a limited viral replication in tissues during acute infection

**Figure 3:**
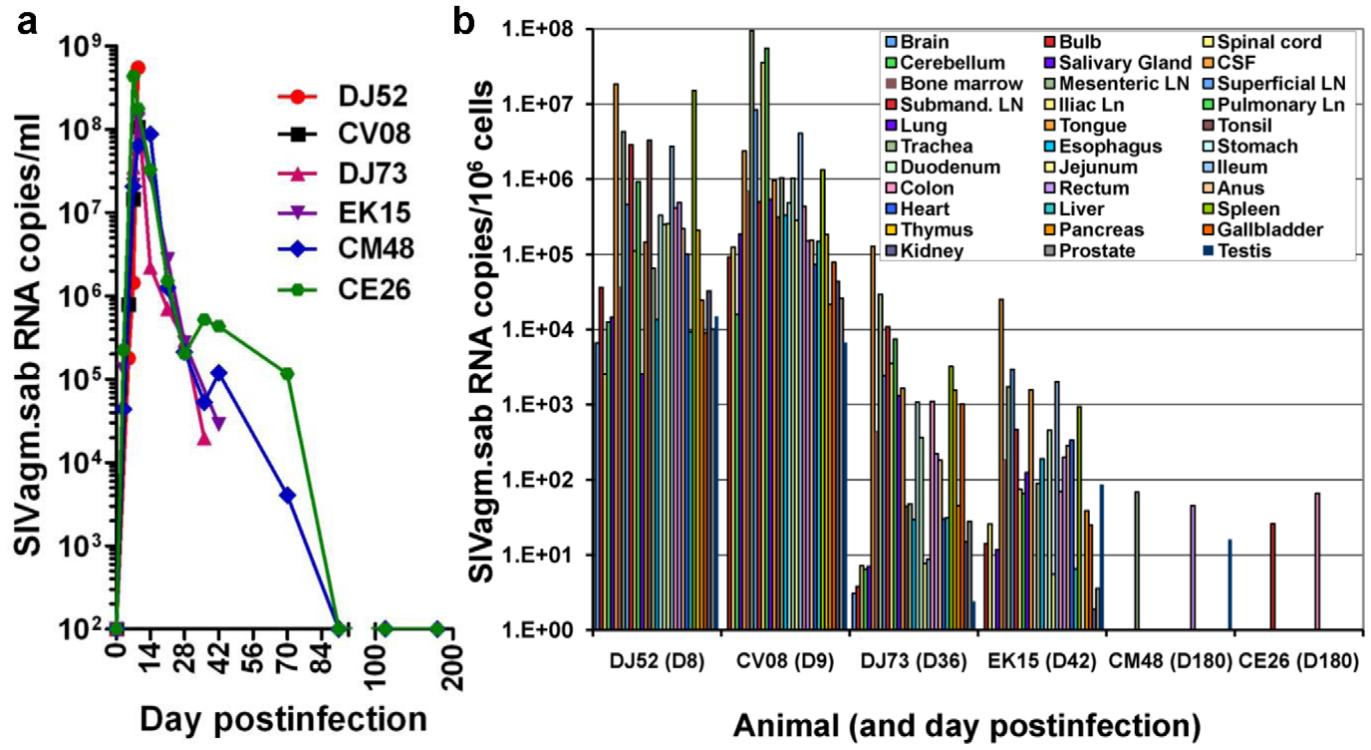
Measurement of SIV RNA in serially sacrificed RMs infected with SIVagm.sab. Six rhesus macaques were included in this study and were serially sacrificed at the peak of viral replication (day 9 pi-RMDJ52 and D10 pi-RMCV08), at the set-point (day 35 pi-RMDJ73 and D42 pi-RMEK15) and during chronic infection (D180pi), when infection is controlled (RMCM48 and RMCE26). (a) Quantification of plasma viral load showed that all these RMs replicated the virus at high levels during acute infection and that the virus load was controlled below detection limits in the RMs euthanized during chronic infection. (b) Quantitative RT–PCR analysis of SIVagm.sab RNA from over 30 tissues obtained at necropsy demonstrated that virus control is not due to a restriction of virus replication to a limited number of anatomical sites.

A viral replication restricted to certain anatomical sites was reported in the past to occur during SHIV infection [25]. In such a pathogenic scenario, CXCR4-tropic SHIVs would predominantly replicate in lymphoid tissues resulting in a very active acute infection followed by the control of viral replication during the chronic stage of infection [26]. To assess the extent of SIVagm.sab replication in RMs, we performed serial necropsies at the peak of viral replication (Days 9 and 10 p.i.), at the set-point (Days 35 and 42 p.i.) and during early controlled infection (Day 180 p.i.). Plasma viral loads are presented in Figure 3a. SIVagm.sab was then quantified on snap frozen tissue fragments collected at the necropsy. RNaseP was used to quantify the number of cells in each tissue. As shown in Figure 3b, during acute infection the virus was present at very high levels in both lymphoid and nonlymphoid tissues. Viral loads dropped by 3–4 logs at the set point (Figure 3b), while during the chronic infection viral replication was generally controlled in all the analyzed tissues, with very low blips being observed in the mesenteric lymph node, rectum and testis in RM CM44 and in the submandibular LN and colon in RM CE26 (Figure 3b). These very low levels of residual tissue viral replication may explain why the residual increased immune activation may persist after the control of plasma VL below detection limits in SIVagm-infected RMs. Similar widespread SIVmac replication was previously reported in RMs, albeit in a more limited number of tissues [27], [28].

The generalized, massive acute viral replication in SIVagm-infected RMs demonstrates that control of viral replication during chronic infection is not due to a preferential viral replication to certain anatomical sites.

### The control of SIVagm.sab replication in RMs does not appear to be due to specific MHC profiles that may drive infection to EC status

All SIVagm-infected RMs were MHC-typed and no correlation between a given MHC type and control of SIVagm replication could be found for RMs (data not shown). With the exception of RM V492, which harbored a B*17 allele, none of the remaining RMs harbored alleles usually associated with control of SIVmac infection (A*01, B*08 or B*17) [19], [20]. Mamu-A*02 allele, a relatively frequent allele in RMs which is not associated with SIV control, was present in 60% of our RMs. We therefore concluded that the control of SIVagm replication in RMs is independent of MHC types.

### Control of SIVagm.sab is not due to an inability of the virus to efficiently infect CD4^+^ T cells in different tissue compartments

**Figure 4:**
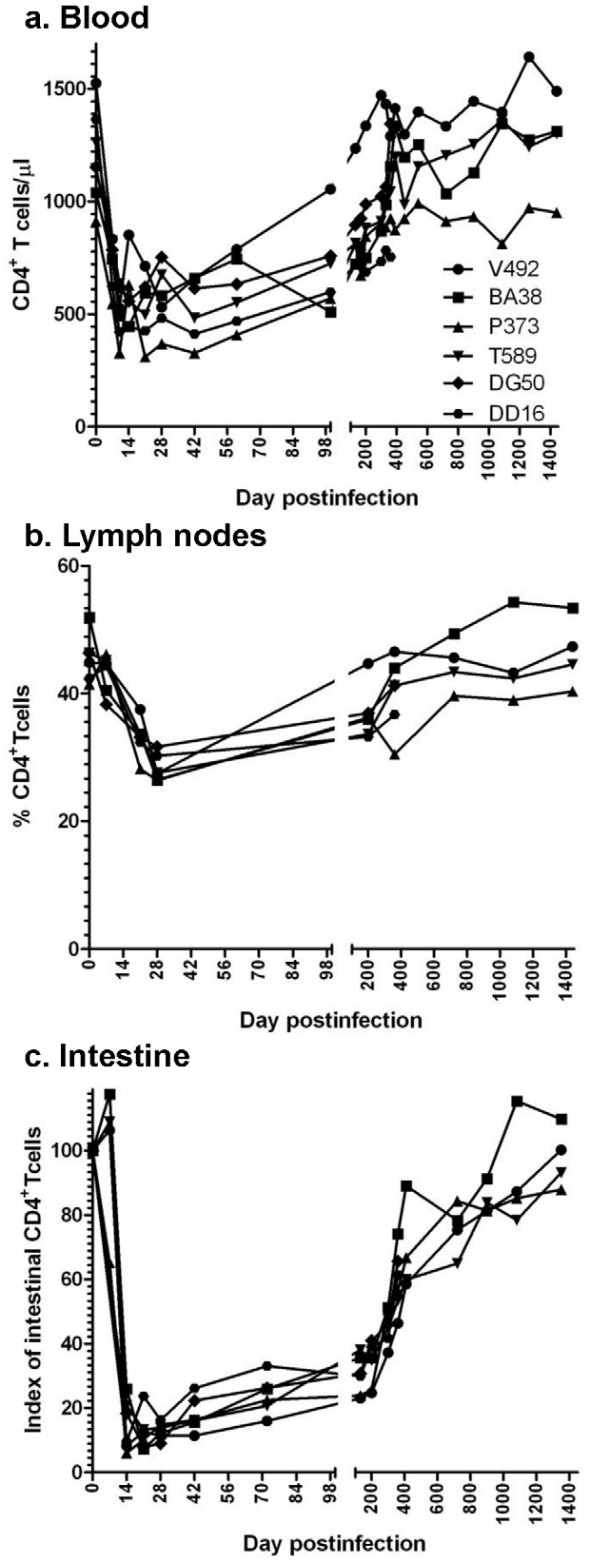
Changes in CD4^+^ T cells in SIVagm-infected RMs. Acute viral replication resulted in a significant depletion of CD4^+^ T cells in blood (a), lymph nodes (b) and intestine (c), which was massive at mucosal sites. With the control of viral replication, complete recovery of CD4+ T cell was observed during the follow-up.

CD4^+^ T cell dynamics were investigated in peripheral blood, lymph nodes (LNs) and intestine of SIVagm.sab-infected RMs using both flow cytometry and immunohistochemistry (IHC). The dynamics of blood CD4^+^ T cells were consistent with the pattern of viral replication. Thus, the high SIV VLs observed during acute infection were accompanied by a moderate (≈30–40%) but significant (p = 0.03) peripheral CD4^+^ T cell depletion after the VL peak (Figure 4a). During the chronic phase, peripheral CD4^+^ T counts rebounded to preinfection levels starting from day 200 p.i. on (Figure 4a), in contrast to pathogenic infections. In the LNs, CD4^+^ T cell depletion was also moderate and persisted up to 200 days p.i.; both flow cytometry (Figure 4b) and IHC on serial LN samples (data not shown) showed complete restoration at late time points.

A dramatic (up to 95%) acute depletion of CD4^+^ T cells occurred by days 14–28 p.i. at the immune effector sites in the lamina propria of the intestine (Figure 4c). IHC on serial samples confirmed marked gut-associated lymphoid tissue (GALT) CD4^+^ T cell loss during primary SIVagm infection of RMs (Figure S2). During chronic SIVagm infection, after the control of SIVagm.sab replication, a gradual immune restoration was observed in RMs (Figure 4c). IHC confirmed this gradual GALT CD4^+^ T cell recovery (data not shown), which resulted in a complete restoration of the mucosal CD4^+^ T cells after 4 years p.i. The mucosal CD4^+^ T cell recovery was incomplete in SIVagm-infected RMs that were followed for 450 days p.i. However, a similar trend for mucosal CD4^+^ T cell recovery was observed in these animals (Figure 4c). This pattern of CD4^+^ T cell changes observed in SIVagm-infected RMs is unprecedented and significantly differs from SIVsmm/mac-infected RMs that show continuous, persistent loss of mucosal CD4^+^ T cells during chronic infection and progression to AIDS [25]. Therefore, we concluded that (i) the outcome of SIVagm infection of RMs is not due to its inability to infect mucosal CD4^+^ T cells; (ii) GALT CD4^+^ T cell depletion during acute infection of SIVagm-infected RMs was of the same order of magnitude as the CD4^+^ T cell depletion that usually occurs in pathogenic SIVsmm infections of RMs, thus not being predictive for clinical outcome of SIV infection; (iii) the complete control of SIVagm infection in RMs resulted in a slow but complete restoration of CD4^+^ T cells.

### Control of SIVagm replication in RMs does not result from a peculiar *in vivo* biology of the virus

We then addressed the question of whether or not the control of SIVagm.sab infection in RMs is due to a particular biology of the virus that may restrict infection to particular CD4^+^ T cell subsets. We have previously characterized SIVagm.sab and showed no significant deletion in any of the accessory genes (particularly the *nef* gene) that might be associated with impaired viral replication [29], [30]. Moreover, in numerous comparative pathogenesis studies in AGMs and pigtailed macaques, we have shown robust levels of *in vivo* replication of SIVagm.sab, as well as progression to AIDS in pigtailed macaques [21], [29], [31] [Pandrea, unpublished].

**Figure 5:**
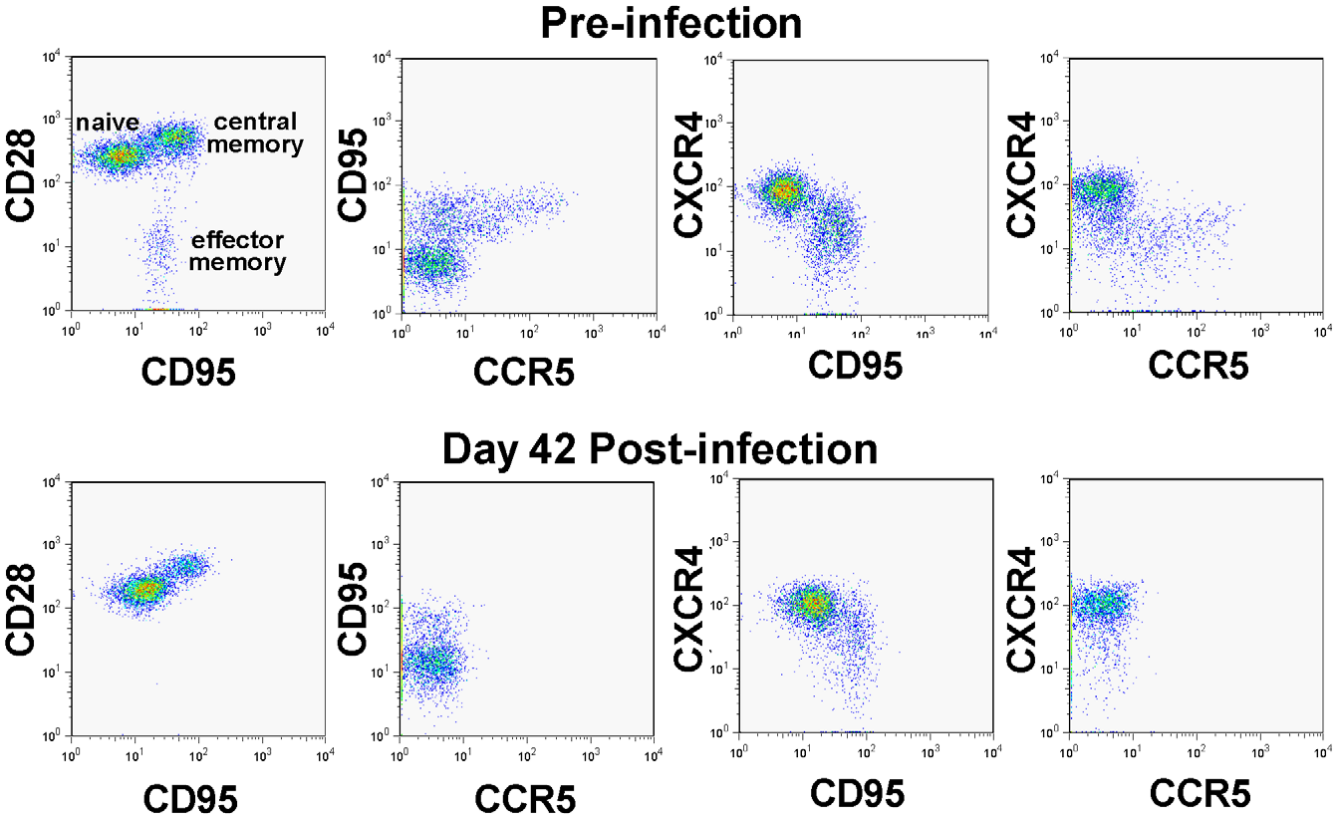
CD4^+^ T cell depletion pattern in SIVagm-infected macaques. PBMCs were examined by polychromatic flow-cytometry for their correlated expression of cell surface CD3 *vs* CD4 *vs* CCR5 *vs* CD28 *vs* CD95 *vs* CXCR4 both before and 28 days after infection. Note the striking depletion of CD4^+^ memory T cells (CD95^+^), and within the memory population, the effector memory (CD28^neg^CD95^+^) and the CCR5-expressing subsets. Meanwhile, CXCR4-expressing cells are well maintained during acute SIVagm.sab infection in RMs. This pattern is identical to the one reported by Picker *et al.,* for CCR5 tropic viruses [25].

We have previously reported, based on *in vitro* studies, that SIVagm.sab92018 has the ability to use CXCR4 in addition to CCR5 as a coreceptor for virus entry [29]. Note, however, that the in vivo dynamics of SIVagm.sab infection in its natural host, the AGM, is very similar to other infections produced by CCR5-tropic SIVs that infect vervets, SMs and mandrills [32], [33]. We therefore investigated whether or not the *in vivo* biology of SIVagm in RMs has particularities that may explain virus control and established multiple lines of evidence that SIVagm.sab is CCR5 tropic *in vivo*: (i) during acute infection of RMs, SIVagm.sab depleted up to 95–99% of CD4^+^ T cells in the intestine (Figure 4c), in agreement with the high CCR5 expression on CD4^+^ T cells at the mucosal sites. Such a massive depletion clearly involved all the CD4^+^ T cell subsets (data not shown). This pattern was similar to all other CCR5-tropic SIVs and HIVs [25] and differed from the *in vivo* pathogenicity of the CXCR4-tropic SHIVs, which show only minimal depletion of mucosal CD4^+^ T cells [25]. (ii) *In vivo*, SIVagm.sab depleted CCR5^+^CD4^+^ T cells and not CXCR4^+^CD4^+^ T cells in macaques, suggesting the *in vivo* usage of CCR5 as the coreceptor of SIVagm in RMs (Figure 5). In addition, phenotyping of the SIVagm-infected cells in RMs also showed that the *in vivo* SIVagm.sab replicated predominantly in lymphocytes in intestine and LNs (Figure S3), very similar to SIVmac [34]. In addition, the effector memory CD4^+^ T cell subset was the cell population most depleted during the SIVagm infection in RMs (Figure 5). We therefore concluded that SIVagm has an *in vivo* biology that is similar to pathogenic lentiviruses able to induce AIDS in NHPs.

We further confirmed these results by assessing the coreceptor usage of the replicating SIVagm during the acute stage of infection (day 10 p.i.) of RMs by testing plasma collected at the peak of viral replication in RMs and comparing it to that observed for the virus in the supernatant of RM PBMC cultures. SIVmac239 (R5-tropic) and SHIV 162P3 (R5-tropic) were used as controls. This experiment confirmed our previous results [29], and showed that *in vitro* passaged SIVagm.sab is dual tropic (CCR5 and CXCR4), similar to other SIVagm.sab strains (Figure S4). However, SIVagm.sab from the plasma during acute infection (i.e., the replicating virus) used only CCR5 as a coreceptor (Figure S4). Therefore, these experiments demonstrate that the control of SIVagm.sab in RMs is not due to a particular biology of the virus.

### CD4^+^ T cell depletion occurred in SIVagm-infected RMs through the same mechanisms as in pathogenic SIVmac infection of RMs

**Figure 6:**
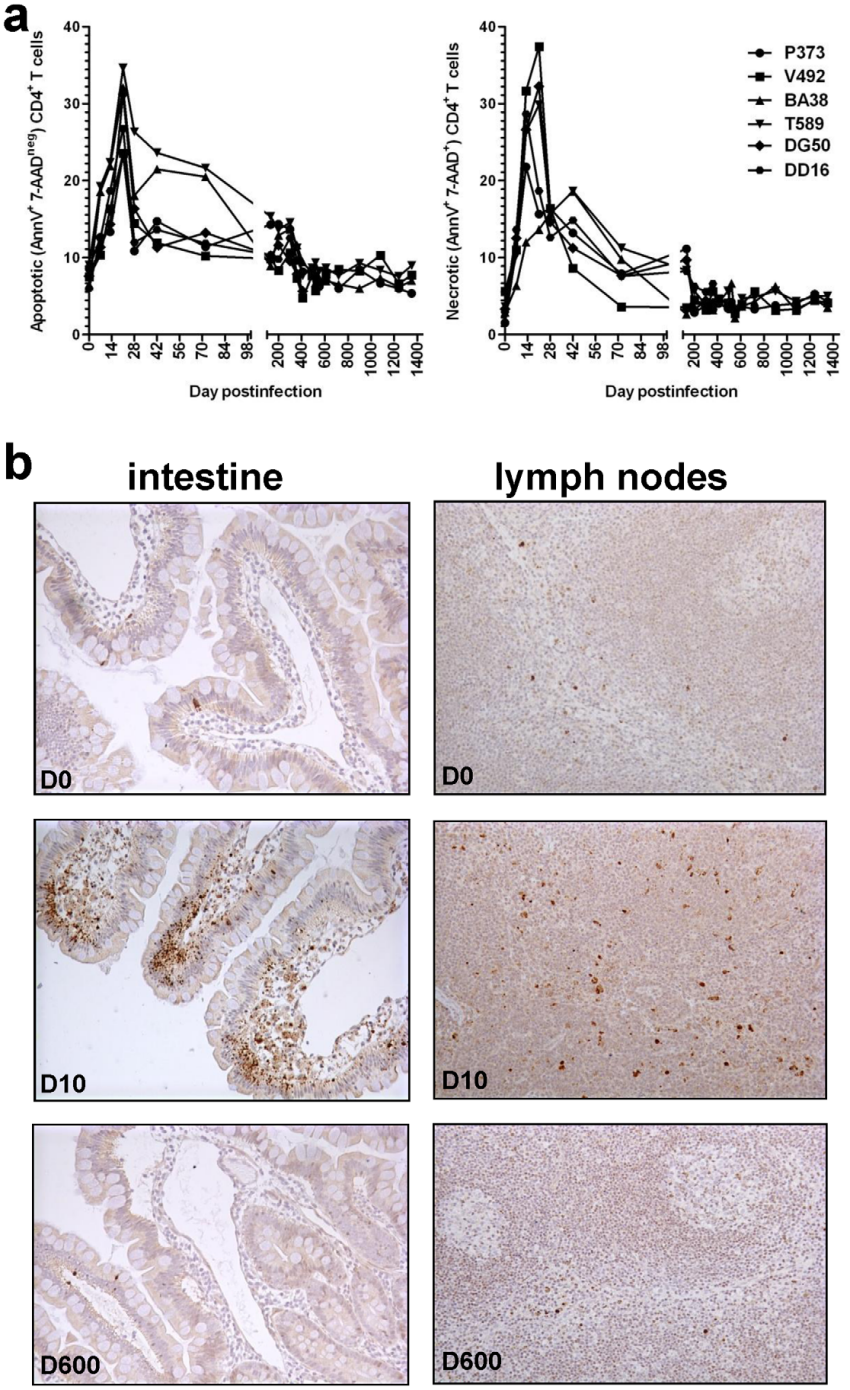
Dynamics of CD4^+^ T cell apoptosis during acute and controlled SIVagm.sab infection of rhesus macaques. (a) Flow cytometric assessment of apoptotic and necrotic cells in the gut showed significant increases of both apoptotic and necrotic cells in association with the high levels of acute viral replication. Increases in apoptosis levels persisted longer than SIVagm viremia being probably responsible for the delays in CD4^+^ T cell restoration. (b) Immunohistochemistry for activated caspase-3. There is a significant increase in apoptosis during the acute infection in both intestine and LNs (middle panels) in SIVagm-infected RMs compared to the pre-inoculation samples from the same sites (upper panels). During late chronic infection the number of activated caspase-3 positive cells decrease in intestine and LNs (lower panels) to levels similar to the pre-infection ones, confirming the flow cytometry data. Note that, during acute SIVagm infection, both lamina propria and intestinal epithelia of RMs show an increased number of apoptotic cells (middle panel, left) thus explaining the transient bacterial translocation.

One of the major differences between pathogenic and nonpathogenic SIV infections relies on the mechanisms of CD4^+^ T cell depletion. In pathogenic infection, destruction occurs through direct killing and indirect mechanisms, such as bystander apoptosis [34]. Conversely, in nonpathogenic infections, the levels of apoptosis do not significantly change during SIV infection [32]. We therefore assessed the mechanisms of CD4^+^ T cell destruction in SIVagm-infected RMs by flow cytometry and IHC. Flow cytometric analysis revealed increases of both necrosis and apoptosis of GALT CD4^+^ T cells that paralleled viral replication (Figure 6a). These results were confirmed by IHC for active caspase-3 that showed increased apoptosis in both intestine and LNs during acute SIVagm.sab infection (Figure 6b, middle panels) compared to preinfection levels (Figure 6b, upper panels) and late chronic infection (Figure 6b, lower panels). These increases in apoptosis persisted after the plasma VLs became undetectable, suggesting bystander apoptosis as a significant contributor to the delay of several months in the restoration of CD4^+^ T cells after viral control (Figure 3). Therefore we concluded that the mechanism of CD4^+^ T cell destruction during acute SIVagm infection of RMs was through both direct viral lysis and bystander apoptosis, being similar to that reported for pathogenic SIVmac-infected RMs [34]. Moreover, the correlation between the normalization of bystander apoptosis and mucosal CD4^+^ T cell recovery during the chronic stage of SIVagm.sab infection in RMs suggests that the main mechanism of CD4^+^ T cell destruction during the chronic infection is through bystander apoptosis.

### The complete control of SIVagm replication in RMs resulted in the restoration of the mucosal immunologic barrier

Microbial translocation from the intestinal lumen to the general circulation may result in increased immune activation and contribute to the CD4^+^ T cell depletion. This event occurs as a result of damage to the mucosa during SIV/HIV infection. During the acute SIVagm infection of RMs, we observed both high viral replication and increases in apoptosis levels of both CD4^+^ T cells and intestinal epithelial cells (Figure 6b), we assessed the dynamics of microbial translocation in RMs by measuring the levels of sCD14 [35]. sCD14 testing was preferred over the LPS testing because it was reported to be a robust test to measure microbial tranlocation [35], while plasma LPS measurements are less reliable (they show 25% interassay variability) [35]. Also, serum LPS levels depend on other factors (i.e., levels of LPS binding, degradation).

**Figure 7:**
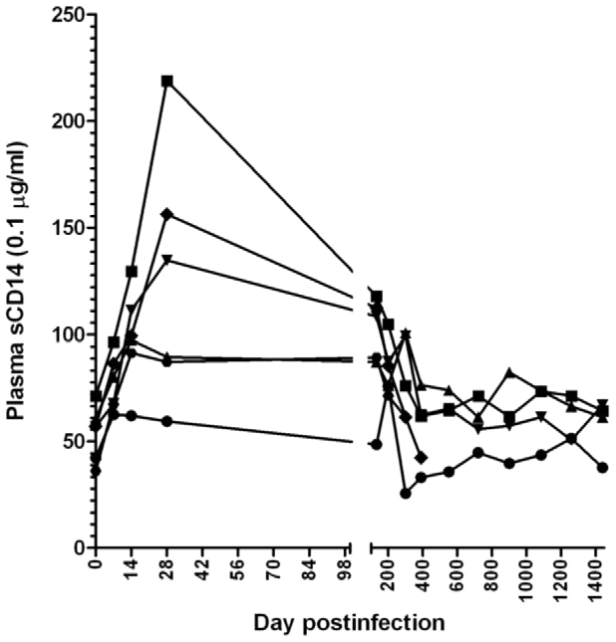
Longitudinal analysis of the microbial translocation in 5 SIVagm-infected RMs. Only a transient increase in plasma levels of sCD14 was observed in SIVagm-infected RMs. Then, with the control of apoptosis, the sCD14 levels returned to baseline.

The massive SIVagm replication during acute infection of RMs led to high levels of immune activation and increases in apoptosis resulted in damage to the intestinal barrier as illustrated by the transient increase in plasma sCD14 (Figure 7). However, after the onset of control of viral replication and normalization of immune activation and apoptosis, sCD14 levels returned to baseline values which were maintained during the follow-up (Figure 7). Therefore, we concluded that if SIV replication is completely controlled, the integrity of the mucosal barrier can be restored and the immune system, although “crippled” by the acute infection, does not progress to “exhaustion” during the chronic phase, as in pathogenic HIV/SIV infections [7].

### The complete control of SIVagm replication is associated with control of T cell immune activation and proliferation in SIVagm-infected RMs

**Figure 8:**
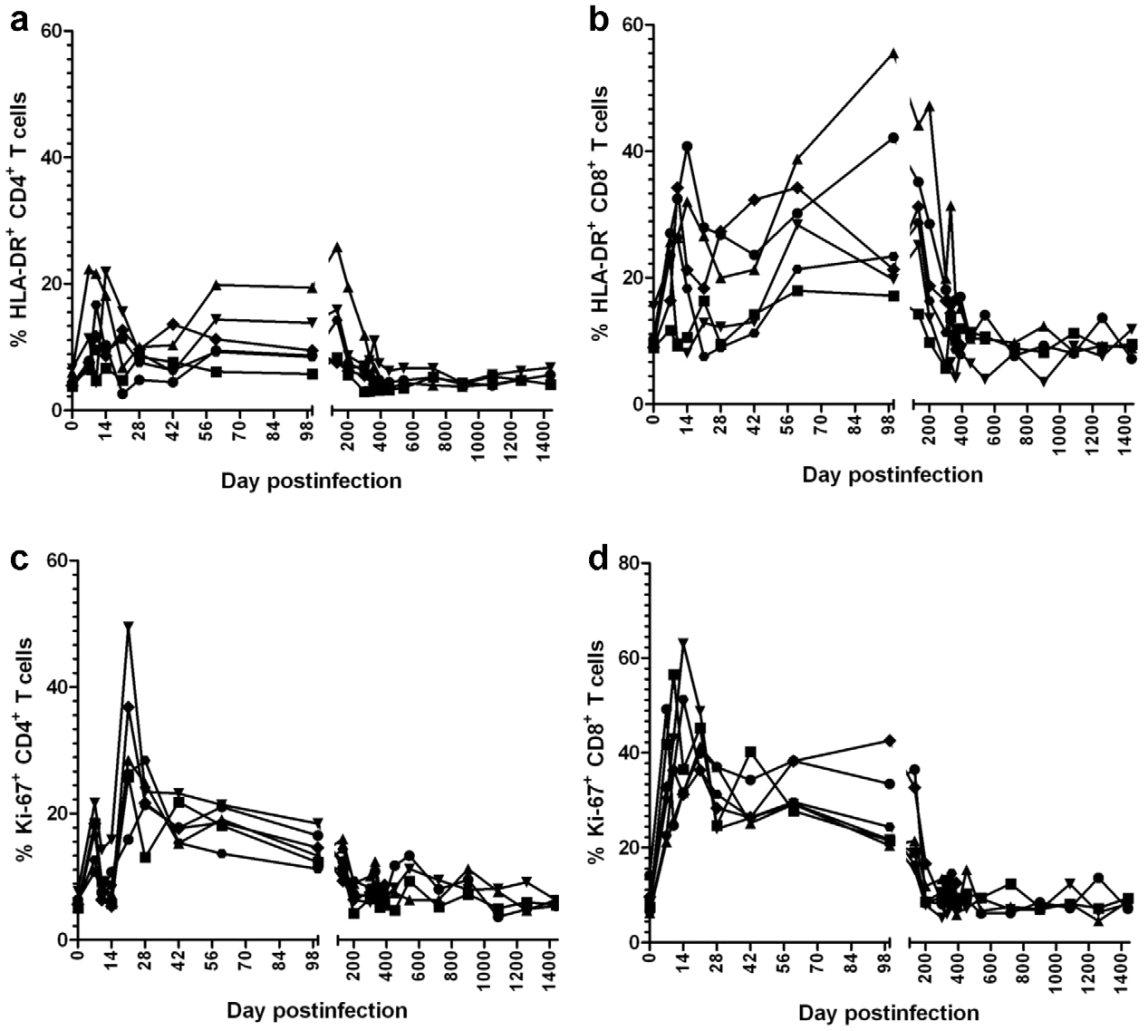
Dynamics of peripheral T cell activation, as assessed by changes in HLA-DR and Ki-67 expression on CD4^+^ and CD8^+^ T cells in SIVagm-infected RMs. SIVagm infection of RMs induced transient levels of activation and proliferation of both CD3^+^ CD4^+^ and CD3^+^ CD8^+^ T cells. After the control of viral replication in tissues, both activation and proliferation markers returned to preinfection levels.

During the acute SIVagm infection of RMs, increases in immune activation (HLA-DR) and cell proliferation (Ki-67) were observed for both CD4*^+^* and CD8*^+^* T cells (Figure 8). These levels started to decrease after the peak of infection, but immune activation and proliferation remained increased for approx. 200 days p.i., paralleling the increased levels of apoptosis (Figure 6). Indeed, the decrease in apoptosis levels and proliferation during this period were highly correlated (r = 0.87, p = 0.016). Overall, both markers of proliferation and apoptosis were highly correlated with CD4^+^ T cell levels in GALT (p<0.0001). Later on, both T cell activation/proliferation (Figure 8) and apoptosis (Figure 6) returned to baseline, which corresponded to significant GALT CD4^+^ T cell restoration (Figure 2). These profiles correlated with those of sCD14 and differed from those observed in pathogenic infections, in which continuous viral replication is associated with persistent damage of intestinal mucosa resulting in continuous and rampant immune activation and cell proliferation, which are associated with disease progression [25]. Therefore, we concluded that (i) the complete control of SIVagm viral replication resulted in normalization of T cell immune activation, proliferation, programmed cell death and subsequent restoration of CD4^+^ T cells to normal levels; (ii) the control of immune activation, cell proliferation and apoptosis occurred after plasma VLs became undetectable and explains delays observed in CD4^+^ T cell restoration; (iii) normalization of T cell immune activation, proliferation and apoptosis provide further evidence for the complete control of SIVagm replication in RMs.

### Control of SIVagm.sab replication in RMs does not appear to be due to host restriction factors

Lentiviruses can infect new host species [32] in spite of the formidable barriers imposed by host defenses. In these cases, a transient viremia may be due to host immune defenses. However, host restriction factors (i.e., APOBEC) might play a role in the control of cross-species transmitted infection through catastrophic GA mutations during reverse transcription that either results in a nonfunctional cDNA or in a cDNA that is targeted for degradation [36]. Therefore, we have investigated the possibility that cross-species transmission of SIVagm.sab to RMs was controlled by a partial host restriction through APOBEC. We have sequenced *env* of SIVagm.sab sampled at different time points from RMs and compared sequence variability to that observed in SIVagm-infected AGMs. Results are shown in Figure S5. No significant accumulation of GA hypermutations was observed in SIVagm-infected RMs. We also evaluated the accumulation of synonymous (*ds*) versus nonsynonymous (*dn*) substitutions in SIVagm strains infecting RMs and AGMs. Evaluation of the *ds/dn* ratio gives an indication of the type of selection pressure that contributes to the evolution of viral sequences. A majority of synonymous mutations (*ds/dn*>1) would indicate the predominance of a purifying type of selection, which is associated with a preferential elimination of viruses with variant amino acids. Conversely, a majority of nonsynonymous mutations (*ds/dn*<1) reflects the predominance of a diversifying type of selection. A comparison between *ds/dn* ratios showed no significant differences between SIVagm-infected RMs (average *ds/dn* ratio: 7.1677; range: 2.13–10.78) and SIVagm-infected AGMs (average *ds/dn* ratio: 6.89; range: 1.98–9.96).

It was recently reported that TRIM5 suppresses cross-species transmission of SIV and that TRIM5 genotype correlate with 100-fold to 1,000-fold differences in viral replication levels [37]. Therefore, we investigated the impact of TRIM5 on the levels of SIVagm.sab replication in RMs as a potential host restriction mechanism of viral replication and we report that no such correlation could be established. SIVagm-infected RMs belonged to different TRIM5 genotypes, such as: TFP/TFP (P373); TFP/Q (BA38; EK15); TFP/CypA (DJ52; CV08; DG50) and Q/CypA (DD16). No difference in viral replication could be observed between these groups.

**Figure 9:**
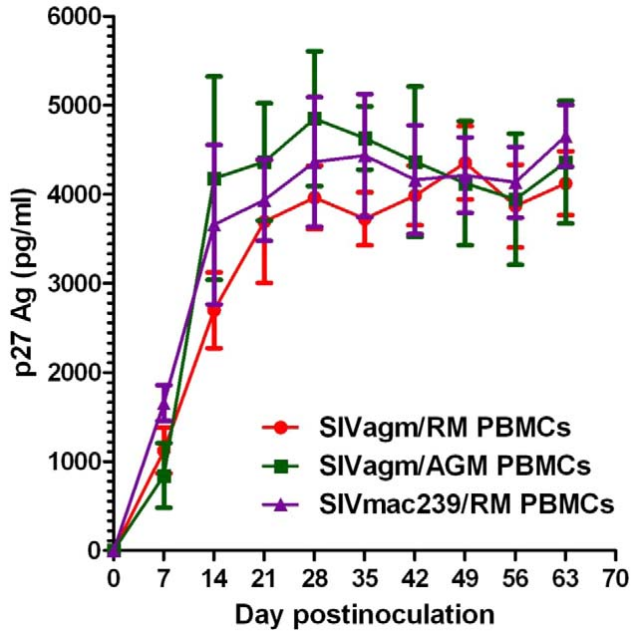
In vitro SIVagm replication on PBMCs from rhesus macaques and AGMs. In vitro replication was tested on PBMCs originating from 3 RMs (DV11, DF93 and DD51). Each experiment was performed in triplicate. In vitro growth curves (±standard deviation) are shown.

Finally, we performed an *in vitro* study of SIVagm.sab92018 replication in RM cells. As controls, we used SIVagm.sab replication in African green monkey PBMCs, as well as the highly pathogenic SIVmac239 on RM PBMCs. Our results showed that SIVagm.sab replicated in peripheral blood mononuclear cells (PBMC) from RMs at similar levels as in AGM PBMCs (Figure 9), in agreement with previous reports [38]. There was a robust and persistent SIVagm *in vitro* replication in both CD4^+^ T cells and monocyte-derived macrophages (MDMs) from AGMs, RMs and humans (data not shown). Persistent SIVagm replication on RM PBMCs, (Figure 9) suggests that the virus is not controlled by host restriction factors.

### Elite controlled SIVagm infection in RMs can be reverted by depletion of CD8^+^ cells, which suggest involvement of immune responses in the EC of viral replication

**Figure 10:**
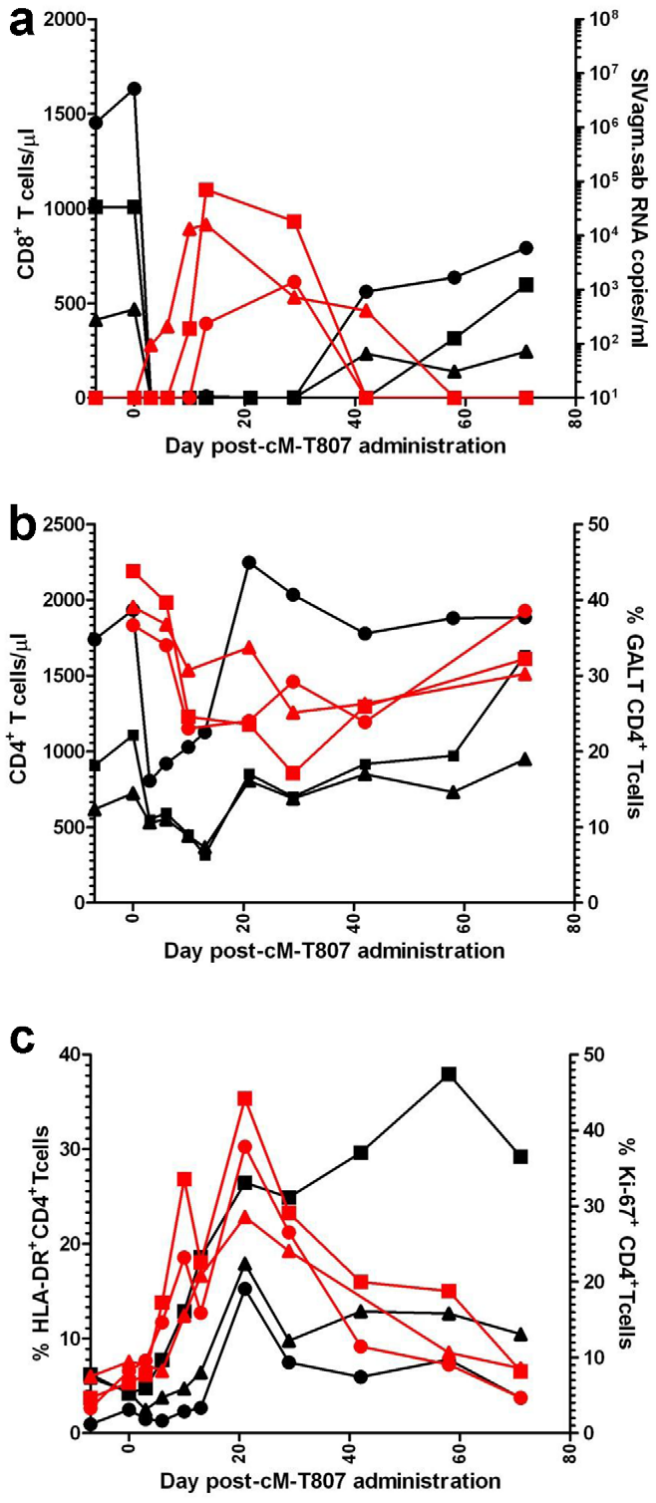
Administration of cM-T807 depleting antibody resulted in a rebound of plasma VLs in all three SIVagm-infected RMs. (a) cM-T807 successfully depleted anti-CD8 cells in periphery and resulted in a rebound of viral replication; (b) transient CD4^+^ T cell depletion in periphery and intestine was observed following the rebound of viral replication (c) increased in CD4^+^ T cell activation and proliferation (as assessed by the study of –DR and Ki-67) were observed, that lasted longer than the rebound in viral replication. CD8 depleted animals: BA38 (▴); V492 (▪); P373 (•).

To ascertain if the lack of detectable VLs in SIVagm-infected RMs is due to control by cellular immune responses, we treated three long-term infected RMs with a single 50 mg/kg dose of cM-T807 anti-CD8 depleting antibody. Two EC SIVagm-infected RMs were sampled according to the same schedule and were used as controls. CD8 depletion was successful for 28–42 days in the periphery in all treated RMs (Figure 10a) and induced downregulation of CD8^+^ T cells in the intestine (not shown). Prior to CD8 depletion, viral replication was undetectable for 4 years (Figures 1 and 2). Postdepletion, a rebound in plasma VLs was observed in 3/3 RMs (BA38, P373 and V492) (Figure 10b). By day 42 post-cM-T807 administration, VLs became undetectable in all RMs. VL control was coincidental with the rebound of CD8^+^ T cells (Figure 10a and b). Viral replication was accompanied by depletion of CD4^+^ T cells in blood and in intestine (Figure 10b). Interestingly, peripheral CD4^+^ T cell depletion was abrupt and preceded the rebound of viral replication (Figure 10b), probably as a consequence of the use of an anti-CD8α depleting antibody [39]. Indeed, a fraction of CD4^+^ T cells that also express the CD8α molecule and may be therefore targeted by the depleting antibody. At the mucosal site, although a significant number of intestinal CD4^+^ T cells express the CD8 molecule, the anti-CD8 antibody only downregulate CD8^+^ cells [39] and therefore mucosal CD4^+^ T cell depletion paralleled the rebound of viral replication (Figure 10b). In all three RMs, the administration of anti-CD8 antibody significantly increased CD4^+^ T cell activation, as defined by -DR expression in periphery (Figure 10c) and intestine (not shown) as well as CD4^+^ T cell proliferation, as defined by Ki-67 expression (Figures 10c). Note that the dynamics of VLs correlated with the CD8^+^ T cell depletion and rebound and not with increased CD4^+^ T cell activation and proliferation, which persisted longer than CD8^+^ T cell depletion. Therefore, we concluded that SIVagm replication appears to be controlled in RMs at least in part by cellular immune responses.

## Discussion

In this study, we showed that rhesus macaque infection with SIVagm results in an infection pattern that models that of elite controlled HIV infection in 100% of cases. Acute SIVagm infection of RMs was similar to pathogenic infection, being characterized by robust acute viral replication and massive mucosal CD4^+^ T cell depletion. However, during the chronic stage of infection, SIVagm was eventually completely controlled in all RMs in blood and tissues. Inflammation and apoptosis were resolved at mucosal sites, microbial translocation was controlled, immune activation returned to baseline levels and mucosal CD4^+^ T cells were completely restored in RMs infected with SIVagm. We also report that SIVagm elite controlled infection in RMs could be reverted by experimental depletion of CD8^+^ cells, suggesting that, similar to HIV-infected human elite controllers, cellular immune responses are involved in the control of SIVagm infection in RMs. Therefore, this new animal model of elite controlled infection may be used to model the viral and host factors involved in the achievement of long-term control of HIV replication in the absence of antiretroviral therapy, i.e., the “functional cure” of HIV infection.

The use of this new animal model for SIV infection allowed us to address some of the most important open questions of SIV/HIV pathogenesis, which may have immediate implication for the management of HIV-infected patients. First, in agreement with our previous reports [31], [40], [41], our model of functional cure for HIV infection demonstrates that acute mucosal CD4^+^ T cell depletion has no prognostic value for the chronic outcome of infection. Second, we report that residual apoptosis and immune activation in subjects with undetectable plasma VLs are due to an incomplete control of viral replication. In SIVagm-infected RMs, low levels of viral replication persisted in tissues several months after plasma VL became undetectable and prevented the control of the apoptosis, microbial translocation and immune activation, hence the immune restoration was quasi inexistent during this time. Conversely, after the achievement of the complete control of virus replication in tissues, apoptosis and immune activation were resolved and normalization of these parameters was followed complete restoration of the mucosal CD4^+^ T cells. These findings suggest that in human ECs, as well as in patients treated with HAART, incomplete immune restoration and persistent elevated levels of immune activation which are observed in spite of undetectable plasma VLs may be due to very low levels of residual viral replication in tissues [42], [43], calling for a therapeutic control of viral reservoirs to restore and preserve the levels of CD4^+^ T cells [44]. Furthermore, our model demonstrates that only the long-term normalization of immune activation and inflammation may lead to complete immune restoration in HIV-infected patients. In our study, mucosal CD4^+^ T cells restoration only occurred four years after the return of apoptosis and immune activation to near baseline levels. Finally, our study showed that sustained complete control of viral replication may result in seroreversion. Seroreversion was not reported in ECs, but occurred in a patient that achieved the functional cure after being treated with allogeneic CCR5Δ32/Δ32 stem cell transplantation for relapsed acute myeloid leukemia (the “Berlin patient”) [45]. As for the SIVagm-infected RMs, the functional cure in this patient was characterized by lack of disease progression for 45 months in the absence of antiretroviral therapy, CD4^+^ T cell reconstitution to normal values in peripheral blood and at mucosal sites, undetectable HIV RNA and DNA in plasma and tissues and seroreversion [45]. These striking similarities between RMs infected with SIVagm and the “Berlin patient”, which is currently the standard for the functional cure of HIV infection, reinforce the value of our new animal model.

In the currently available animal models of EC infection, control is only achieved in a fraction of monkeys or it is due to significant attenuation of the virus. Thus, viral replication of attenuated strains of SIVmac, such as Δ-nef or Δ-3 strains [46] is impaired in all phases of infection by significant alterations in virus structure. This pattern is in sharp contrast with the majority of the EC infection in humans in which the viruses are replication-competent without major deletions [47]. Second, suppression of viremia in pathogenic macaque infections through highly active antiretroviral treatments [48] does not fulfill the definition criteria for EC infection, as the animals are ARV-treated and low levels of viral replication, immune activation and incomplete immune restoration persist under treatment [48]. Third, during SIVmac infections in Chinese (Ch) RMs long-term nonprogression occurs only in a subset of monkeys [49], cannot be predicted based on *in vitro* testing [50] and is associated with persistent viral replication in tissues [28]. Reagents are not yet fully tested in ChRMs and MHC characterization is incomplete, as opposed to IndRMs. Finally, a fraction of SIVmac239-infected Indian RMs with particular MHC profiles is defined as “elite-controlling” infection [19], [20]. Note, however, that “EC” SIVmac-infected RMs show persistent low levels of viral replication (10^2^–10^3^ copies/ml) [19]. Moreover, as for ChRMs, control of infection cannot be predicted before it is achieved. The advantage of the EC model reported here over the existing models, is that in SIVagm-infected RMs, a robust acute viral replication is followed by complete control in all cases, therefore, to date, this animal model is the only one that can be used for the study of early events of SIV infection leading to EC infection and to define the biomarkers that will allow early identification of the HIV infected patients that have the potential of controlling infection and the delineation of the parameters of a successful functional cure of HIV infection (i.e., control of viral replication in tissues, normalization of apoptosis and immune activation, seroreversion etc).

One may argue that, since SIVagm infection is cross-species transmitted to RMs, the proposed model might not be relevant because control is dependent on host restriction factors. We have several lines of evidence that the role of host intrinsic immunity may be peripheral in achieving control of infection in this model: (i) SIVagm replicated at high levels in RMs during acute infection; (ii) *in vitro* data showed persistent high levels of SIVagm.sab replication (comparable to that of SIVmac) on RM PBMCs; (iii) the analysis of SIVagm evolution in RMs showed no evidence of hypermutation that might have been the result of a partial host restriction through the deaminase system; (iv) the high ds/dn ratios observed in RMs suggest that SIVagm was under purifying selection in the postacute phase of SIVagm.sab infection; finally, (v) Trim5 genotypes of the infected monkeys were not associated with particular viral replication profiles; (vi) CD8 depletion experiments in controllers resulted in rebounds of VL, thus pointing to an immune control of viral replication.

Another critique to this new animal system may be that it only models a fraction of controlled HIV infections. LTNPs are a heterogeneous group of patients, in which different mechanisms may lead to various levels of viral control [1], [51]. Thus, viremic controllers have residual levels of viral replication. Elite controllers, on the other hand, control viral replication to undetectable levels of plasma viral load, but the majority of ECs have persistent levels of increased immune activation [52], [53], inflammation-associated vascular dysfunction [54] and declining CD4 counts over time [52], [53]. Only a fraction of EC patients (the “super-elites”), achieve control of immune activation close to baseline levels, in addition to the control of viral replication. These patients sustain CD4^+^ T cell counts and preserved CD8^+^ T cell function [Landay, unpublished] and are probably the best examples of functional cure of HIV infection. Even if these patients represent a minority of the HIV infected patients, understanding the mechanisms through which the functional cure of HIV infection occurs in the absence of antiretroviral therapy is probably the most important information that can be derived from the study of controlled HIV/SIV infections for both vaccine development and HIV eradication efforts. Our new animal system models these super-elite controllers, which are the most difficult to model in animal systems and therefore it is a major achievement in the field, as it can be used to identify the factors driving the infection to elite controlled status overcoming the most important limitation to the study of the functional cure, which is that control cannot be predicted at the time when the virus actively replicate during the early stages of infection.

We conclude that SIVagm infected RMs represent a valuable model of super elite controlled infection which can be used to: (i) examine virologic and immunologic changes during early infection that may lead to the infection control; (ii) perform invasive studies facilitating concomitant investigations in a large array of tissues collected at critical time points of infection; (iii) perform *in vivo* manipulation of SIV pathogenesis by selective depletion of different cellular subsets, or experimental modulation of immune activation to assess their contribution to the control of VL. Such experiments have not been and cannot be pursued in studies of human HIV-1 ECs and thus, this new model addresses an immediate need in AIDS research: deciphering the mechanisms and biomarkers of durable and effective control of SIV replication.

## Materials and Methods

### Ethics statement

The animals were fed and housed according to regulations set forth by the *Guide for the Care and Use of Laboratory Animals* [40] and the Animal Welfare Act. The animal experiments in this study were approved by the Tulane University Institutional Animal Care and Use Committee (IACUC).

### Animals, infection and samples

Twelve male RM (*Macaca mulatta*) aged 5–11 years were used. Animals were intravenously inoculated with plasma equivalent to 150 tissue culture infectious doses (TCID50) of SIVagm.sab92018 [21]. The first group of 6 RMs were followed for up to 6 years p.i. (the pathogenesis group) to characterize the clinical outcome of SIVagm infection, the dynamics of viral replication and the impact of SIVagm infection on the major immune cell populations; the remaining 6 RMs were serially sacrificed at days 9, 10, 35, 42 and 180 p.i. to assess the dynamics and the levels of tissue replication.

### Tissue sampling

Blood was collected from all the animals in the pathogenesis group at 2 time points preinfection (days -30, -7 p.i.), then at the time of SIVagm.sab inoculation, twice per week for the first two weeks p.i., weekly for the next four weeks, every two weeks for the next two months and then every three months, up to 6 years p.i. LN biopsies were sampled on days 0, 8, 28, 200, 400, 800, 1200 and 1400 p.i. Intestinal endoscopies (proximal jejunum) consisting of approximately 10–15, 1–2 mm^2^ pieces were obtained by endoscopic guided biopsy were performed on days 0, 8, 14, 21, 28, 42, 72, 84, 100, and then every three months up to 6 years p.i. Intestinal resections (five to ten cm) were performed at days preinfection (D-30) and at days 10/42 and 200/600 p.i., as previously described [31], [55]. Additional intestine pieces were obtained at necropsy. The serially sacrificed RMs were sampled following the same sampling schedule. Plasma and peripheral blood mononuclear cells (PBMCs) and mononuclear cells from the intestine and LNs were isolated as described [10], [21]. The serially sacrificed RMs were euthanized and up to 38 different tissue samples were collected. Mononuclear cells were separated from all mucosal and lymphatic tissues as described [10], [21], while total RNA was extracted directly from 100 mg of snap frozen parenchimatous tissues.

### Viral quantification

Plasma VLs were quantified as described [29]. Assay sensitivity was 100 RNA copies/ml of plasma. For quantification in tissues, viral RNA was extracted from 5×10^5^–10^6^ cells originating from multiple tissues with RNeasy (Qiagen, Valencia, CA). Snap frozen tissues were ultrasonicated prior to RNA extraction. VLs were quantified in blood and tissues as described [21]. Simultaneous quantification of RNase P (TaqMan Copy Number Reference Assay RNase P, Applied Biosystems, Carlsbad, CA) normalized the RNA input from cells [31]. Assay sensitivity was 10 RNA copies/10^6^ cells.

### Analysis of anti-SIVagm.sab immunoglobulin G responses

An in-house SIVagm.sab-specific primate immunodeficiency virus enzyme immunoassay was used for the titration of anti-gp41 and anti-V3 antibody titers, as described previously [56], on serial plasma or serum samples to investigate the dynamics of anti-SIVagm.sab seroconversion and seroreversion. SIVagm.sab neutralization was measured using a new neutralization assay. An SIVagm.sab-specific molecularly cloned Envpseudotyped virus containing full-length gp160 of SIVagm.sab92018 (clone 28) was prepared as described previously [24]. Neutralization titers were then measured as 50% reductions in luciferase reporter gene expression in TZM-bl cells, as reported previously [24].

### Antibodies and flow-cytometry

Immunophenotyping of lymphocytes isolated from the blood, LNs and intestine was performed by using fluorescently conjugated monoclonal antibodies using a seven-color staining technique. The samples were run using a LSR-II flow cytometer (Becton Dickinson) and the data were analyzed using FlowJo (Tree Star, Inc). The following mAbs were used for flow cytometry: CD3-Pacific blue (clone no. SP34), CD4-APC (clone no. L200), CCR5-PE (clone no. 3A9), HLA-DR-APC-Cy7 (clone no. L243), Ki-67-FITC (clone no. B56) (BD Bioscience), CD95-FITC (clone no. DX2), CD28-PE-Cy7 (clone no. CD28.2) CD8αβ-Texas Red (clone no. 2ST8.5H7) (Beckman Coulter). All antibodies were validated and titrated using RM PBMCs. Samples were stained for apoptosis using Annexin V: PE Apoptosis Detection kit I (BD Pharmingen) as per manufacturer instructions. Apoptotic CD4^+^ T-cells were defined as Annexin V^pos^7AAD^neg^, whereas the necrotic CD4^+^ T-cells were defined as Annexin V^pos^7AAD^pos^. CD4^+^ and CD8^+^ T-cell percentages were obtained by first gating on lymphocytes, then on CD3^+^ T-cells. Memory, activation, proliferation and apoptosis markers were determined by gating on lymphocytes, then on CD3^+^ T cells and finally on CD4^+^CD3^+^ or CD8^+^CD3^+^ T cells.

### Immunohistochemical staining (IHC) and *in situ* hybridization (ISH)

IHC was performed on formalin-fixed, paraffin-embedded tissues using an avidin-biotin complex horseradish peroxidase technique (Vectastain Elite ABC kit, Vector Laboratories, Burlingame, CA) and either mouse monoclonal anti-human CD4 (NCL-CD4-1F6, Novocastra, Newcastle, UK) or rabbit polyclonal Activated Caspase-3 (Abcam, Cambridge, MA). For SIV *in situ* hybridization (ISH), sections were subjected to high-temperature unmasking, treated with 0.2 N HCl, and hybridized overnight at 45°C with either sense or antisense SIVagm digoxigenin-UTP labeled riboprobe, blocked with normal sheep serum, incubated with sheep anti-digoxigenin-alkaline phosphatase (AP) and incubated with Ferangi blue. SIVagm-infected cell phenotype was determined after ISH by incubating sections with: rabbit anti-human CD3 (DAKO, Carpinteria, CA); mouse anti-human macrophage (HAM56; DAKO); followed by the appropriate anti-mouse or anti-rabbit secondary antibodies. ABC method (Vectastain Elite ABC kit) and amino-ethylcarbazole (AEC) (DAKO) were also used to detect HAM56 and CD3. Negative controls included an antisense probe with uninfected tissues, a sense probe with infected tissues, an antisense probe with infected tissues and anti-rabbit or anti-mouse secondary antibodies only.

Microbial translocation was monitored by measuring sCD14 levels, as previously described, using a commercially available ELISA (R&D systems) [35]. Plasma samples were diluted 5 fold with endotoxin-free water and then heated to 70°C for 10 minutes to inactivate plasma proteins. Plasma was diluted 1∶300 and assay was performed in duplicate according to the manufacturer's protocol.

Coreceptor usage was determined as described previously [50]. Human osteosarcoma (GHOST) cells expressing CD4 and one of the following coreceptors were obtained through the NIH AIDS Research and Reference Program, Division of AIDS, AIAID contributed by Dan Littman and Vineet KewalRamani: CCR1, CCR2, CCR3, CCR4, CCR5, CCR8, CXCR4, BOB and Bonzo. These cells were cultured in complete Dulbecco's minimal essential medium containing G418 (5 µg/ml), hygromycin (1 µg/ml), and puromycin (1 µg/ml). GHOST cells expressing only CD4 (GHOST-CD4 cells) served as controls; these cells were cultured in the same medium without puromycin. GHOST cells (10^5^/ml; 500 µl per well) were maintained in 24-well plates for 24 h. The medium was then removed, and 200 µl of fresh medium was added, along with a viral inoculum of 10 MOI. On the next day, residual virus was removed and the cells were washed once with 1 ml of medium. Then, 750 µl of fresh complete medium containing the selection antibiotics was added. Productive viral replication was monitored by measuring SIV P27 Gag antigen in the culture supernatants on days 0, 2, 4, 6, and 9 by ELISA (Zeptometrix Corp., Buffalo, NY). In all cases, the amount of antigen produced in control GHOST-CD4 cells was subtracted from the amount produced in coreceptor-transfected GHOST-CD4 cells.

### SIVagm.sab92018 *env* sequencing

Viral evolution *in vivo* in RMs was investigated as follows: SIVagm RNA was extracted as described above from serial plasma samples (collected between days 10–72 pi) and subjected to PCR amplification. Then, viral RNA was retro-transcribed by using superscript II RNAse H^-^ Reverse Transcriptase (Invitrogen) according to the manufacturer's instructions using primers EnvASab and EnvBSab [29]. Reverse transcription was done at 25°C for 10 min, 50°C for 30 min and 70°C for 15 min. Resulted cDNA was amplified by PCR using the proof-reading DNA polymerases TAKARA Ex Taq (Takara Bio) or Platinum Taq (Qiagen) following the manufacturer's instructions. The PCR conditions consisted of 40 cycles of denaturation (95°C for 10 s), of hybridization (55°C for 30 s) and elongation (72°C for 1 min). A seminested PCR was then performed using the same conditions as for the first round PCR. Primers used were EnvAsab and EnvBsab for the first round and EnvAsab and NS3asVerTYO (5′ GAA GCC TAA GAA CCC TAG CAC AAA 3′) for the seminested reaction [29]. PCR products were visualized by agarose gel electrophoresis. PCR products were cloned using the TOPO TA Cloning Kit (Invitrogen). Plasmids with the correct insert were sequenced with the universal primer and e*nv* sequences were aligned, translated and analyzed.

### TRIM5α sequencing

The TRIM5α genotypes were determined for RM by isolating genomic DNA from PBMCs and directly sequencing the 526 nucleotides PCR product of the B30.2/SPRY domain of TRIM5α. The sequence of the primers utilized both for PCR and for the sequencing reaction are CAGTGCTGACTCCTTTGCTTG for the forward primer and GCTTCCCTGATGTGATAC for the reverse primer. The obtained sequences were characterized by polymorphisms at nucleic acid position 997, 1015–1020, 1022 of TRIM5α.

### Infection of PBMC from different host species

PBMCs were isolated from RM and AGM blood as described earlier [50]. Freshly isolated PBMCs were stimulated with 10 µg PHA per ml of medium for 2 days followed by overnight incubation in IL-2 media. Activated PBMCs (5×10^6^) were infected with SIVagm stocks containing 4,000 pg of P27 at 37°C for 4 h; cells were then washed extensively to remove any cell-free virus. Cells were maintained in IL-2 media for 8 weeks. Virus production in culture supernatants was monitored weekly by SIV P27 antigen capture assay.

### *Anti-CD8 Ab treatments*

Three SIVagm-infected RM controllers were treated intravenously with cM-T807, a mouse anti-human monoclonal anti-CD8 antibody. Treatments consisted of an initial dose of 50 mg/kg followed by administration of 10 mg/kg after 6 and 13 days, respectively. cM-T807 administration was followed by a frequent sampling of blood (days 0, 4, 7, 10, 14, 21, 28, 35, 42, 56, 72 post cM-T807 administration) as well as intestinal biopsies (days 0, 7, 14, 21, 28, 42, 72 post cM-T807 administration).

### Statistical analysis

Data comparisons were done using two-tailed non-parametric tests (Mann-Whitney). Correlation analyses among time-varying variables were done by comparing slopes or using gee with working independence [57]. Analyses were performed using R (R Foundation for Statistical Computing, Vienna, Austria).

## Supporting Information

Figure S1 **High levels of SIVagm replication in lamina propria during acute infection of RMs, as determined by *in situ* hybridization.**
(PDF)

Figure S2 **Immunohistochemical assessment of CD4^+^ T cell depletion during SIVagm infection of rhesus macaques.** Massive CD4^+^ T cell depletion compared to baseline levels (D0) was observed after acute SIVagm infection (D42).
(PDF)

Figure S3 **Immunophenotyping of the SIVagm infected cells in rhesus macaques.** Combined *in situ* hybridization for SIV (blue) and immunohistochemistry (red) for either macrophage (HAM56) (a) or lymphocyte (CD3) (b) markers demonstrates that during the acute SIVagm infection of RMs, the majority of the infected cells are lymphocytes.
(PDF)

Figure S4 **Comparison between co-receptor usage of plasma RNA virus from acutely-infected RMs (DD16, DG50, day 10 p.i.) and the SIVagm.sab passaged in vitro (co-culture with RM PBMCs) as measured at day 9 post-inoculation.** Although the *in vitro* passaged virus appears to be dual (CCR5 and CXCR4) tropic, similar to parental SIVagm.sab92018 (passaged *in vitro* in AGM PBMCs), the plasma virus is exclusively CCR5-tropic. Controls: SIVmac239 and SHIV162P3. Note that, while SIVagm.sab is preferentially using Bonzo, SIVmac is preferentially using Bob.
(PDF)

Figure S5 **Partial gp120 env (678 bp) sequence evolution in AGM EI42 and RMs P373, V492 and BA38 infected with SIVagm.sab.92018.** Three clones were sequenced from each similar time points post-infection, as listed. Sequences of the relapsing virus post-CD8 cell depletion are also shown (in violet). No significant differences in viral evolution were noted between AGM and RMs.
(PDF)
