# Supplementary figures and images for: Functional Cure of SIVagm Infection in Rhesus Macaques Results in Complete Recovery of CD4+ T Cells and Is Reverted by CD8+ Cell Depletion
Source: PLoS Pathog. 2011 Aug 4;7(8):e1002170. doi: 10.1371/journal.ppat.1002170 (PMC3150280; doi:10.1371/journal.ppat.1002170)

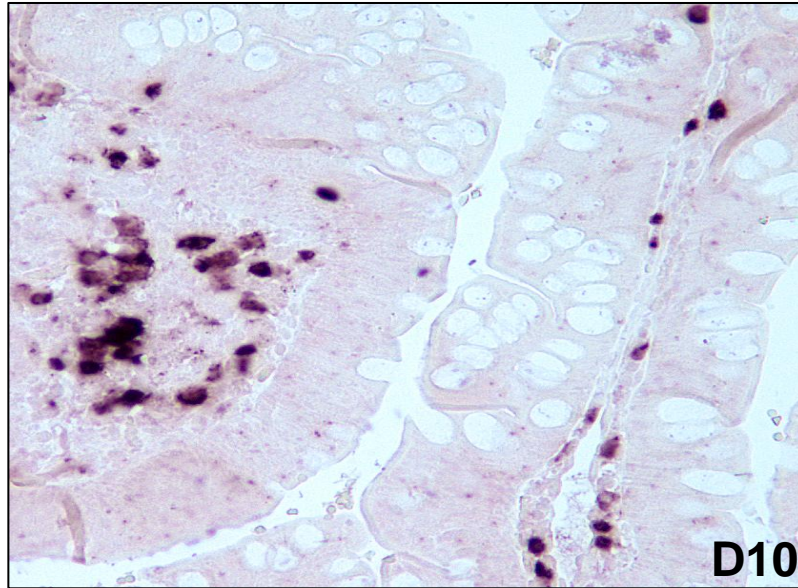

Supplement: Figure S1 — High levels of SIVagm replication in lamina propria during acute infection of RMs, as determined by in situ hybridization. (PDF) [file ppat.1002170.s001.pdf]

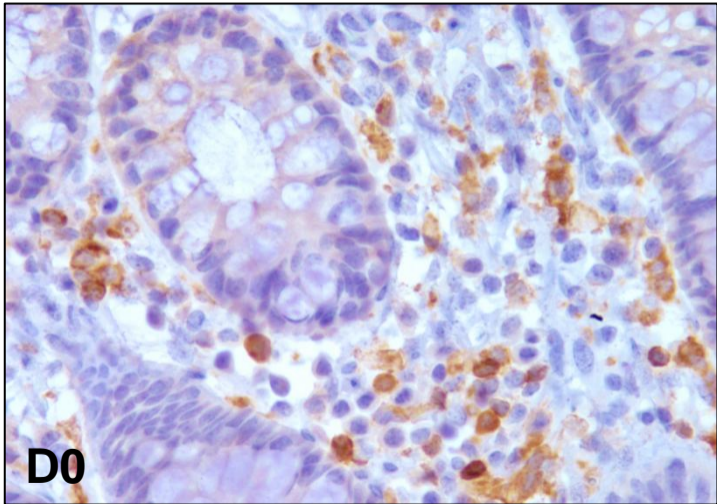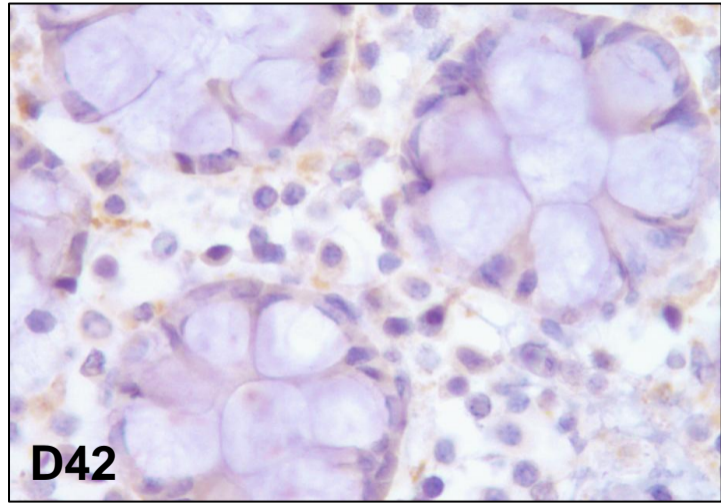

Supplement: Figure S2 — Immunohistochemical assessment of CD4+ T cell depletion during SIVagm infection of rhesus macaques. Massive CD4+ T cell depletion compared to baseline levels (D0) was observed after acute SIVagm infection (D42). (PDF) [file ppat.1002170.s002.pdf]

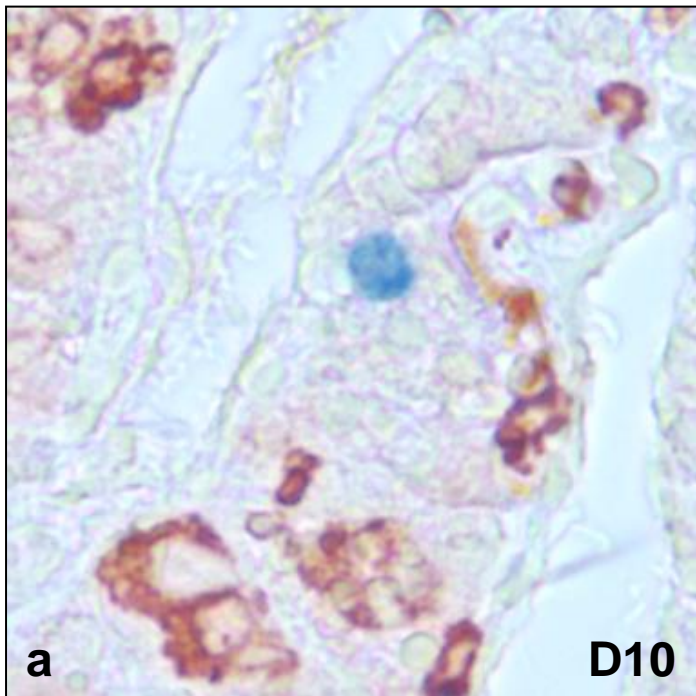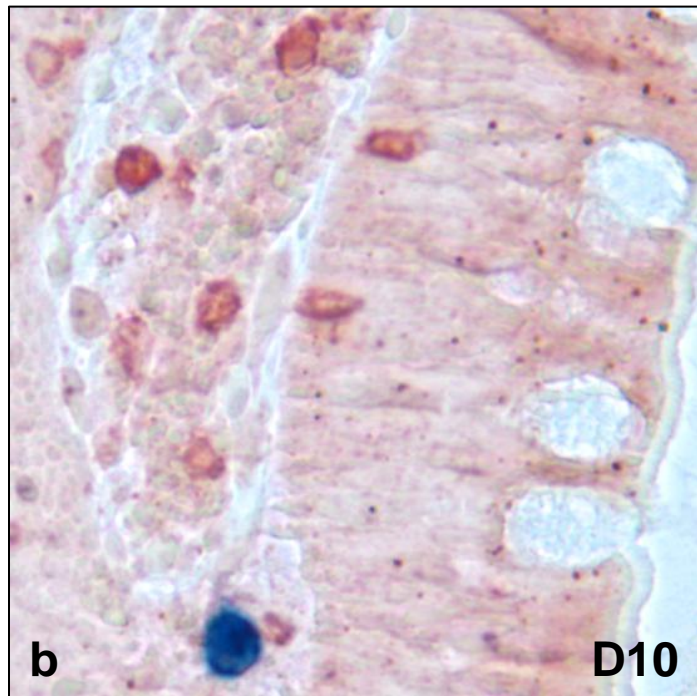

Supplement: Figure S3 — Immunophenotyping of the SIVagm infected cells in rhesus macaques. Combined in situ hybridization for SIV (blue) and immunohistochemistry (red) for either macrophage (HAM56) (a) or lymphocyte (CD3) (b) markers demonstrates that during the acute SIVagm infection of RMs, the majority of the infected cells are lymphocytes. (PDF) [file ppat.1002170.s003.pdf]

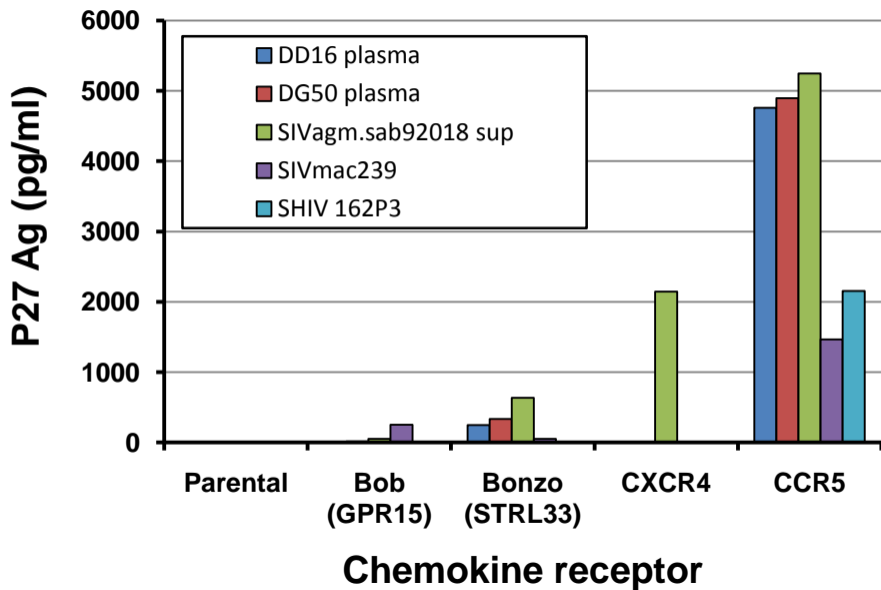

Supplement: Figure S4 — Comparison between co-receptor usage of plasma RNA virus from acutely-infected RMs (DD16, DG50, day 10 p.i.) and the SIVagm.sab passaged in vitro (co-culture with RM PBMCs) as measured at day 9 post-inoculation. Although the in vitro passaged virus appears to be dual (CCR5 and CXCR4) tropic, similar to parental SIVagm.sab92018 (passaged in vitro in AGM PBMCs), the plasma virus is exclusively CCR5-tropic. Controls: SIVmac239 and SHIV162P3. Note that, while SIVagm.sab is preferentially using Bonzo, SIVmac is preferentially using Bob. (PDF) [file ppat.1002170.s004.pdf]
