# Supplementary material for: Functional Cure of SIVagm Infection in Rhesus Macaques Results in Complete Recovery of CD4+ T Cells and Is Reverted by CD8+ Cell Depletion
Source: PLoS Pathog. 2011 Aug 4;7(8):e1002170. doi: 10.1371/journal.ppat.1002170 (PMC3150280; doi:10.1371/journal.ppat.1002170)
Supplement: Figure S5 — Partial gp120 env (678 bp) sequence evolution in AGM EI42 and RMs P373, V492 and BA38 infected with SIVagm.sab.92018. Three clones were sequenced from each similar time points post-infection, as listed. Sequences of the relapsing virus post-CD8 cell depletion are also shown (in violet). No significant differences in viral evolution were noted between AGM and RMs. (PDF) [file ppat.1002170.s005.pdf]

Supplementary Figure 5

```
                   ....|....|....|....|....|....|....|....|....|....|....|....|....|....|....|....|....|....|....|....|....|....|....|
                           10        20        30        40        50        60        70        80        90       100       110
SIVagm.Sab         RRPGNKTVLPVTIMAGLVFHSQKYNTRLKQAWCWFGGNWTQAWKEVRETIVKLPNTTYKGTRNISQIFLQRQWGDPEAENFFFNCQGEFFYCKMDWFLNYLNNQSVDPDHNPCKT
AGM_EI42_D9_3      ...........................................R......................V..........S.D.................................NK
AGM_EI44_D9_1      ..................................................................................................................D
AGM_EI44_D9_2      .....................................S............................................................................D
AGM_EI44_D20_1     ............M................................................................S....................................D
AGM_EI44_D20_2     .................................................................................................................NK
AGM_EI44_D42_1     .............................................................................S....................................D
AGM_EI44_D42_2     ............T....................................................................................................D
AGM_EI44_D93_1     .............................................................................S...................................NK
AGM_EI44_D93_2     .............................................................................S...................................NK

RM_P373_D10_1      ....................C.......................................................................R......................
RM_P373_D10_2      ...................................................................................................................
RM_P373_D10_3      ...................................................................................................................
RM_P373_D21_1      ...................................................................................................................
RM_P373_D21_2      ..................................................................................................................D
RM_P373_D21_3      ...................................................................................................................
RM_P373_D21pd_1    ......................................................D..........................................................GN
RM_P373_D21pd_2    ......................................................D................R....................................G....GN
RM_P373_D21pd_3    ......................................................D..........................................................GN
RM_P373_D21pd_4    ......................................................D................................................R.........GN

RM_V492_D10_1      .............................................................................S...................................GN
RM_V492_D10_2      .......................F.......................................M......................R..........................NK
RM_V492_D10_3      .......................F....................................................................R.....................D
RM_V492_D42_1      .............................................................................S....................................D
RM_V492_D42_2      .............................................................................S....................................D
RM_V492_D42_3      .............................................................................S..S.................................D

RM_BA38_D10_1      ................................................................................................L................NK
RM_BA38_D10_2      ............................................................................................R......................
RM_BA38_D10_3      ...........................................E..K............E......................................................D
RM_BA38_D21_1      ............................................................................................................N....NK
RM_BA38_D21_2      ....................................E.R.......K....................................................................
RM_BA38_D21_3      ...................................................................................................................
RM_BA38_D93_1      .......................F...................................E................................R.N....................
RM_BA38_D93_2      .......................F...................................E................................R.N.............G......
RM_BA38_D93_3      .......................F...................................E................................R.N....................
RM_BA38_D21pd_1    ..............................................................S............................................S.......
RM_BA38_D21pd_2    ..............................................................S....................................................
RM_BA38_D21pd_3    ..............................................................S....................................................
RM_BA38_D21pd_4    ..............................................................S....................................................
```

```
                    ....|....|....|....|....|....|....|....|....|....|....|....|....|....|....|....|....|....|....|....|....|....|....
                        120       130       140       150       160       170       180       190       200       210       220
SIVagm.Sab          KGDKKCWQRTYVPCHIRQVVNDWYTLAKKTYAPPREGHLECTSTATALYVELNYNSNNRTNVTLSPQIRSIWSYELGDYKLVEIKPIGFAPTEVRRYTGPERQKRVPFVLGFLG
AGM_EI42_93         T.......................................................H.........................................................
AGM_EI44_9_1        ..................................................................................................................
AGM_EI44_9_2        ...........................................M......................................................................
AGM_EI44_20_1       ........................................................................P.........................................
AGM_EI44_D20_2      T.................................................................................................................
AGM_EI44_42_1       ..........................................A..................................G....................................
AGM_EI44_42_2       ....................................................................................................K.............
AGM_EI44_93_1       T.................................................................................................................
AGM_EI44_93_2       T.................................................................................................................

RM_P373_D10_1       ..................................................................................................................
RM_P373_D10_2       .............................A....................................................................................
RM_P373_D10_3       ..................................................................................................................
RM_P373_D21_1       ..................................................................................................................
RM_P373_D21_2       ........................................................................................L.........................
RM_P373_D21_3       ..................................................................................................................
RM_P373_D21pd_1     ...................................................F................................................K.............
RM_P373_D21pd_2     ...................................................F................................................K.............
RM_P373_D21pd_3     ...................................................F................................................K.............
RM_P373_D21pd_4     ....R....................................A.........F................................................K.............

RM_V492_D10_1       ..................................................................................K.................K.............
RM_V492_D10_2       T...R.............................................................................................................
RM_V492_D10_3       ..................................................................................................................
RM_V492_D42_1       ..................................................................................................................
RM_V492_D42_2       ..................................................................................................................
RM_V492_D42_3       ..................................................................................................................

RM_BA38_D10_1       T.................................................................................................................
RM_BA38_D10_2       ..................................................................................................................
RM_BA38_D10_3       ..................................................................................................................
RM_BA38_D21_1       T.................................................................................................................
RM_BA38_D21_2       ..................................................................................................................
RM_BA38_D21_3       ...............................................P..................................................................
RM_BA38_D93_1       ..................................................................................................................
RM_BA38_D93_2       ..................................................................................................................
RM_BA38_D93_3       ..................................................................................................................
RM_BA38_D21pd_1     ..................................................................................................................
RM_BA38_D21pd_2     ..................................................................................................................
RM_BA38_D21pd_3     ..................................................................................................................
RM_BA38_D21pd_4     ..................................................................................................................
```
